# Clinical characteristics, prognostic factors, and maternal and neonatal outcomes of SARS‐CoV‐2 infection among hospitalized pregnant women: A systematic review

**DOI:** 10.1002/ijgo.13329

**Published:** 2020-08-30

**Authors:** Ozlem Turan, Amir Hakim, Pradip Dashraath, Wong Jing Lin Jeslyn, Alison Wright, Rezan Abdul‐Kadir

**Affiliations:** ^1^ Department of Obstetrics and Gynecology Royal Free Hospital NHS Trust London UK; ^2^ EGA Institute for Women’s Health University College London London UK; ^3^ National Heart and Lung Institute Imperial College London London UK; ^4^ Barts and the London School of Medicine and Dentistry Queen Mary University of London London UK; ^5^ Department of Obstetrics and Gynecology Yong Loo Lin School of Medicine National University of Singapore Singapore

**Keywords:** COVID‐19, Intrauterine fetal demise, Maternal morbidity, Maternal mortality, Miscarriage, Neonatal morbidity, Neonatal mortality, Preterm birth

## Abstract

**Background:**

Pregnant women represent a potentially high‐risk population in the COVID‐19 pandemic.

**Objective:**

To summarize clinical characteristics and outcomes among pregnant women hospitalized with COVID‐19.

**Search strategy:**

Relevant databases were searched up until May 29, 2020.

**Selection criteria:**

Case series/reports of hospitalized pregnant women with laboratory‐confirmed COVID‐19.

**Data collection and analysis:**

PRISMA guidelines were followed. Methodologic quality was assessed via NIH assessment tools.

**Main results:**

Overall, 63 observational studies of 637 women (84.6% in third trimester) with laboratory‐confirmed SARS‐CoV‐2 infection were included. Most (76.5%) women experienced mild disease. Maternal fatality, stillbirth, and neonatal fatality rates were 1.6%, 1.4%, and 1.0%, respectively. Older age, obesity, diabetes mellitus, and raised serum D‐dimer and interleukin‐6 were predictive of poor outcomes. Overall, 33.7% of live births were preterm, of which half were iatrogenic among women with mild COVID‐19 and no complications. Most women underwent cesarean despite lacking a clear indication. Eight (2.0%) neonates had positive nasopharyngeal swabs after delivery and developed chest infection within 48 hours.

**Conclusions:**

Advanced gestation, maternal age, obesity, diabetes mellitus, and a combination of elevated D‐dimer and interleukin‐6 levels are predictive of poor pregnancy outcomes in COVID‐19. The rate of iatrogenic preterm birth and cesarean delivery is high; vertical transmission may be possible but has not been proved.

## Abstract 2

Perinatal and maternal morbidity and mortality are increased in severe/critical COVID‐19 disease; there is a high rate of iatrogenic preterm birth and cesarean delivery.

## 1 INTRODUCTION

Coronavirus disease 2019 (COVID‐19) is caused by severe acute respiratory syndrome coronavirus 2 (SARS‐CoV‐2) and was first reported in Wuhan, Hubei Province, China, in December 2019. The infection has spread swiftly across the globe to 188 countries, affecting over thirteen million individuals with a case fatality rate of 5%, and was declared a pandemic by the WHO on March 11, 2020.^1^

The initial data on COVID‐19 outcomes in pregnancy were derived from small numbers of patients, first from China and then from Italy, with uncertainty regarding the extent to which these findings might be extrapolated to inform obstetric practice elsewhere in the world. Small international data sets have subsequently emerged, but these are often from different institutions with heterogeneous testing policies, obstetric management, and patient populations. Population‐level studies are therefore urgently required to provide robust data on the incidence of COVID‐19 in pregnancy and its effects on the pregnant woman, developing fetus, and newborn.

The objective of the present review was to evaluate clinical characteristics and maternal, fetal, and neonatal outcomes among pregnant women admitted to hospital with laboratory‐confirmed SARS‐CoV‐2 infection by conducting a large global comprehensive review of data from various epicenters. By addressing practical issues surrounding the parameters that predict prognosis across the disease spectrum, the review aims to inform clinical practice and guide policy in maternity services for this and future global pandemics affecting pregnant women and those wishing to conceive.

## 2 MATERIALS AND METHODS

The present systematic review was conducted in accordance with Preferred Reporting Items for Systematic Reviews and Meta‐Analyses (PRISMA) guidelines.^2^ PubMed, Ovid Medline, Web of Science, and China Academic Literature Database were searched for studies on pregnant women with COVID‐19 infection from database inception until May 29, 2020. The search strategy combined terms for SARS‐CoV‐2, COVID‐19, pregnancy, maternal mortality, maternal morbidity, complications, miscarriage, preterm birth, neonatal morbidity, intrauterine fetal demise, and neonatal mortality. Studies from mainland China were included. Three studies published in Mandarin were translated into English by two of the researchers (JWJL and PD).^3^, ^4^, ^5^ Studies in languages other than English or Mandarin were excluded.

The review included case series and case reports of pregnant women with SARS‐CoV‐2 infection confirmed by either quantitative real‐time polymerase chain reaction (RT‐PCR) or dual fluorescence PCR assessment. Unpublished reports, articles in which the date and location of the study were not specified, women with suspected COVID‐19 that was not confirmed by laboratory tests, and studies that did not report maternal, fetal, or neonatal outcomes were excluded.

Studies were independently retrieved and reviewed for eligibility by two authors (OT and RK). The following data were extracted: Severity of COVID‐19, gestational age at presentation, presenting symptoms, laboratory parameters, admission to intensive care unit (ICU), need for Invasive mechanical ventilation (IMV), gestational age at delivery, mode of delivery, admission to NICU, stillbirth, neonatal death and maternal death.

Data analysis, including prevalence and incidence levels, was performed by using SPSS version 26.0 (IBM, Armonk, NY, USA). Categoric variables were expressed as number (percentage).

The methodologic quality of studies was assessed by using National Institutes of Health quality assessment tools.^6^ Each study was classified as low (≥7), moderate (5–6), or high risk of bias (≤4).

## 3 RESULTS

### 3.1 Included studies

**Figure 1:**
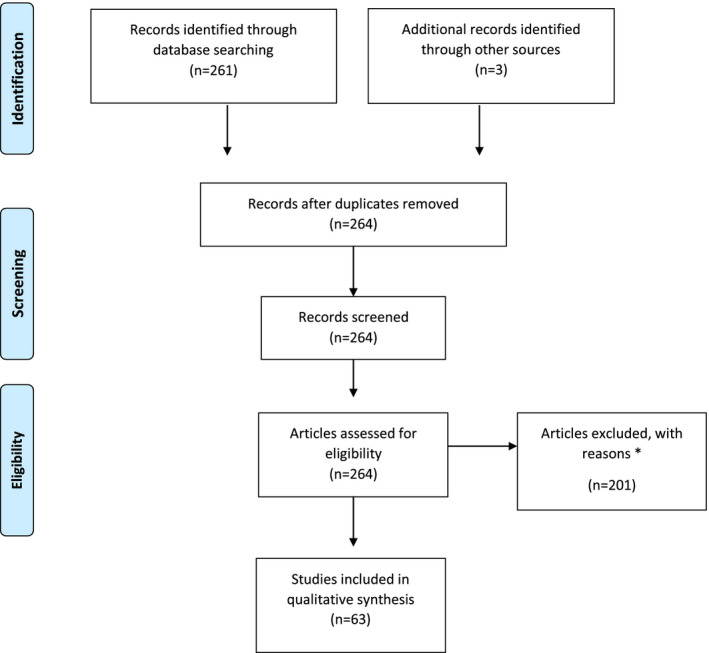
PRISMA flowchart showing selection of studies for review. The reasons for excluding studies were (1) type of study (only case reports, case series, and retrospective cohort analyses were included); (2) language (non‐English and non‐Mandarin publications were excluded); (3) access to full text (abstract‐only publications were excluded); and (4) confirmation of SARS CoV‐2 infection (only laboratory‐confirmed cases were included).

In total, 261 articles were identified in the initial search from March 26 to May 29, 2020; of these, 63 observational studies met the inclusion criteria (Fig. 1). There were 28 case series, 31 case reports, and 4 retrospective cohort studies. All 63 observational studies were eligible for qualitative synthesis (Fig. 1).

Overall, the mean risk for publication bias was moderate (6.4 out of 9) across all case series; 4 (14%) and 8 (29%) of case series reported high or moderate risk of publication bias, respectively. Subgroup analysis indicated that a high percentage of case series (n=25; >85%) adequately described the research questions and study population, and ensured that study subjects were comparable. Only 12 (43%) and 15 (54%) of case series fully described statistical methods and patient results, respectively. Consequently, reporting of statistical methods and full analysis of study data posed the greatest risk of publication bias.

### 3.2 Clinical characteristics

The clinical characteristics of all 63 observational studies are documented in File S1. In total, 637 pregnant women were admitted to hospital with laboratory‐confirmed SARS‐CoV‐2 infection on RT‐PCR tests, of whom 636 women were diagnosed via nasopharyngeal swabs and 1 via broncho‐alveolar lavage. On admission, 32 (5.0%), 55 (8.6%), and 539 (84.6%) women were in the first, second, and third trimester of pregnancy, respectively (Table 1). The gestational age on admission was not reported for 11 women in one study.^7^

**Table 1:** Adverse maternal outcomes stratified by trimester and COVID‐19 severity.

Outcome	Total no. of women	Trimester^a^			COVID‐19 severity		
		1st	2nd	3rd	Mild	Severe	Critical
ICU admission							
No. of women assessed	637	32	55	539	487	101	49
Outcome observed	61	1 IMV	5 IMV	45 IMV 10 NIMV 4 ECMO^b^		12	49
Hemorrhage							
No. of women assessed	637	35	55	525	487	101	49
Outcome observed	4 APH 6 PPH			4 APH 6 PPH	4 APH 2 PPH	3 PPH	1 PPH
Venous/arterial thromboembolism							
No. of women assessed	637	35	55	525	487	101	49
Outcome observed	3			3		1	2
Abnormal clotting/DIC							
No. of women assessed	637	35	55	525	487	101	49
Outcome observed	6	0	0	6		0	3 AC 3 DIC
Maternal death							
No. of women assessed	637	35	55	525	487	101	49
Outcome observed	10		2	8			10

Abbreviations: AC, abnormal clotting (prolonged partial thromboplastin and activated partial thromboplastin times); APH, antepartum (prepartum) hemorrhage; DIC, disseminated intravascular coagulopathy; IMV, invasive mechanical ventilation; PPH, postpartum hemorrhage; TOP, termination of pregnancy; TPL, threatened preterm labor.

^a^ Trimester of pregnancy was not specified for 11 women in one case series (8). None of these women had reported ICU admission, haemorrhage, VTE, abnormal clotting/ DIC or maternal death.

^b^ Extra‐corporeal membrane oxygenation treatment required ultimately after IMV.

Overall, 487 (76.5%), 101 (15.9%), and 49 (7.7%) were diagnosed on admission as having, respectively, mild, severe, and critical COVID‐19 disease, according to clinical criteria defined by the Chinese Centre for Disease Control and Prevention.^8^ Medical co‐morbidities were reported for 359 women.^7^, ^9^, ^10^, ^11^, ^12^, ^13^, ^14^, ^15^, ^16^, ^17^, ^18^, ^19^, ^20^, ^21^, ^22^, ^23^, ^24^, ^25^, ^26^, ^27^, ^28^, ^29^, ^30^, ^31^, ^32^, ^33^, ^34^, ^35^ The most common co‐morbidity was overweight or obesity (n=118, 32.9%), bronchial asthma (n=37, 10.3%), essential hypertension (n=21, 5.8%), and type 2 diabetes mellitus (n=10, 2.8%). Seven (1.9%) women were aged 40 years or older. Presenting symptoms were reported in 55 studies, comprising 515 pregnant women.^4^, ^5^, ^7^, ^11^, ^12^, ^13^, ^14^, ^15^, ^16^, ^17^, ^18^, ^19^, ^21^, ^22^, ^23^, ^24^, ^25^, ^26^, ^27^, ^28^, ^29^, ^30^, ^32^, ^33^, ^34^, ^35^, ^36^, ^37^, ^38^, ^39^, ^40^, ^41^, ^42^, ^43^, ^44^, ^45^, ^46^, ^47^, ^48^, ^49^, ^50^, ^51^, ^52^, ^53^, ^54^, ^55^, ^56^, ^57^ Of these, the most common symptoms at presentation were fever (n=273, 53.0%), cough (n=224, 43.5%), myalgia/malaise (n=79, 15.3%), dyspnea (n=64, 12.4%), diarrhea (n=18, 3.5%), and sore throat (n=18, 3.5%), regardless of gestational age. Four women presented with reduced fetal movements and three with prepartum hemorrhage. Notably, 68 (10.7%) women in 10 studies were asymptomatic on admission.^4^, ^5^, ^12^, ^13^, ^21^, ^37^, ^41^, ^53^, ^55^, ^64^

Serum biochemistry results were reported in 37 studies encompassing 381 women respectively.^4^, ^5^, ^9^, ^10^, ^11^, ^13^, ^14^, ^15^, ^17^, ^18^, ^20^, ^21^, ^25^, ^29^, ^30^, ^33^, ^34^, ^35^, ^36^, ^39^, ^41^, ^44^, ^45^, ^46^, ^47^, ^48^, ^49^, ^50^, ^53^, ^56^, ^57^, ^58^, ^59^, ^60^, ^61^, ^62^, ^63^, ^66^ In this group, raised serum C‐reactive protein was reported among 275 (72.2%) women, lymphopenia among 179 (47.0%) women, and transaminitis among 93 (24.4%) women. In addition, leukopenia and leukocytosis were equally observed among 53 women (13.9%) each. Raised serum D‐dimer was reported among 94 (24.7%) women, including 30 with a mild disease. A combination of raised serum D‐dimer and interleukin‐6 (IL‐6) levels was reported for 68 (17.8%) women, of whom 4 had mild, 44 had severe and 20 had critical COVID‐19. Notably, this represented approximately 60% and 80% of the subgroup of women with severe and critical disease, respectively.

Chest imaging findings were reported in 42 studies (n=274 women), and comprised 267 computed tomography (CT) scans of the chest, 15 plain chest X‐rays, and 5 CT pulmonary angiogram (CTPA) examinations.^4^, ^5^, ^9^, ^10^, ^11^, ^13^, ^15^, ^16^, ^17^, ^22^, ^23^, ^24^, ^25^, ^26^, ^27^, ^28^, ^29^, ^30^, ^33^, ^34^, ^35^, ^36^, ^37^, ^39^, ^40^, ^41^, ^44^, ^45^, ^49^, ^50^, ^51^, ^53^, ^57^, ^59^, ^60^, ^62^, ^63^, ^64^, ^65^, ^66^ In 26 studies, totaling 221 women from China with mild COVID‐19, 196 (88.7%) had abnormal chest CT and X‐ray findings, of whom 17 (8.7%) were asymptomatic at the time of radiologic evaluation.^4^, ^5^, ^9^, ^10^, ^11^, ^13^, ^16^, ^29^, ^34^, ^35^, ^36^, ^37^, ^40^, ^41^, ^44^, ^45^, ^49^, ^50^, ^51^, ^53^, ^56^, ^57^, ^58^, ^59^, ^60^, ^62^, ^63^, ^64^ Abnormal pulmonary findings among pregnant women with mild disease included focal unilateral or bilateral ground glass opacities, whereas diffuse, bilateral ground glass opacities with subpleural involvement and pleural effusion were observed among all women with severe and critical COVID‐19. There were two instances of pulmonary embolism diagnosed among the five CTPA examinations recorded in the included studies.

### 3.3 Maternal outcomes

The incidence of adverse maternal outcomes was assessed for all 637 pregnant women (Table 1). The rate of intensive care unit (ICU) admission was 9.6% (n=61) (Table 1) and comprised 1 (1.6%) women in the first trimester, 5 (8.2%) women in the second trimester, and 55 (90.2%) women in the third trimester of pregnancy.^7^, ^15^, ^18^, ^24^, ^25^, ^26^, ^27^, ^28^, ^29^, ^30^, ^31^, ^42^, ^44^, ^48^, ^53^, ^59^, ^63^, ^66^ Among the 487 women admitted with mild COVID‐19, 9 (1.8%) deteriorated to severe or critical disease necessitating escalation of care to the ICU. Of the 68 women who were initially asymptomatic at presentation, 2 (2.9%) later developed respiratory distress and were admitted into the ICU with critical disease. Invasive mechanical ventilation (IMV) was required for 51 (83.6%) of the 61 women in the ICU,^7^, ^15^, ^18^, ^24^, ^25^, ^26^, ^27^, ^28^, ^29^, ^30^, ^31^, ^32^, ^42^, ^44^, ^48^, ^50^, ^59^, ^66^ and 10 (16.4%) were managed with non‐invasive mechanical ventilation^7^, ^50^, ^53^, ^63^; 4 women ultimately required extra‐corporeal membrane oxygenation.^34^, ^47^, ^49^, ^55^ Among the 61 women admitted to the ICU, 10 women were reported to have died, 1 was still on extra‐corporeal membrane oxygenation, 4 remained intubated, and 46 had been successfully extubated.

Overall, the incidence of maternal mortality was 1.6% (10/637); all of these women were admitted to the ICU with severe or critical COVID‐19 requiring IMV.^15^, ^26^, ^59^, ^66^ The number of maternal deaths accounted for 16.4% (10/61) of all ICU admissions and 20.4% (10/49) of women with critical disease. Maternal death occurred in the second trimester for two women; the remaining eight women were admitted in the third trimester and died between 0 and 18 days postpartum. Two of the 10 women who died were 40 years or older^15^; two women had diabetes mellitus and obesity.^15^, ^26^

Hemorrhagic complications were observed in 10 cases (Table 1). Four women presented at hospital with antepartum hemorrhage described as one of their presenting symptoms.^45^, ^51^ In addition, six women experienced postpartum hemorrhage (estimated blood loss >1000 mL), all of whom had undergone cesarean delivery. Three of these women had severe or critical COVID‐19: one had developed consumptive coagulopathy requiring treatment with fibrinogen concentrate, whereas the other two had mild COVID‐19 disease.^18^, ^20^, ^32^

Six women had abnormal coagulation parameters, all of which were associated with critical infection and multi‐organ dysfunction; three of three women subsequently died.^18^, ^20^, ^44^, ^59^ Coagulation parameters returned to normal during the postpartum period among the three women who survived.

Three thrombotic events were reported among the study women included in the review. All three events occurred despite the administration of thromboprophylaxis with low molecular weight heparin. One woman was admitted with mild COVID‐19 symptoms at 29 gestational weeks^60^; however, she deteriorated rapidly and developed acute respiratory failure that required continuous positive airway pressure therapy. She did not have pre‐existing co‐morbidities except obesity or a family history of thrombophilia or venous thromboembolism. The second women, aged 29 years, was admitted at 31 gestational weeks with mild COVID‐19 symptoms and had diabetic ketoacidosis with a background medical history of obesity, insulin‐dependent diabetes mellitus, renal tubular acidosis, and bronchial asthma.^26^ After rapid respiratory compromise, she was admitted to the ICU for IMV. Subsequent CT head and CTPA confirmed right lower lobe pulmonary embolus and basilar artery thrombosis. As a consequence of the grave cardiopulmonary and neurologic prognosis, treatment was withdrawn and she died. The third women was critically ill in the second trimester of pregnancy with multiple arterial line thromboses, possibly predisposed by her twin pregnancy and pre‐existing chronic inflammatory disease.^30^ She survived and was discharged still pregnant from hospital with regular follow‐up at the time of reporting. The use of low molecular weight heparin thromboprophylaxis was described only in one case series including 27 pregnant women with severe and 20 with critical COVID‐19; all women received thromboprophylaxis and none developed thrombotic complications.^18^

Four studies reported 14 placental histopathology results, all showing evidence of occlusive fibrin deposition and placental hypoperfusion with non‐occlusive thrombi.^45^, ^52^, ^54^, ^55^ Notably, all placentae were from women with clinically mild COVID‐19, among whom there were seven preterm deliveries, one small for gestational age neonate, one case of placental abruption, and one case of second‐trimester miscarriage.

### 3.4 Fetal and neonatal outcomes

Miscarriage or termination of pregnancy was reported in six studies.^32^, ^33^, ^34^, ^53^, ^54^, ^63^ In total, there were seven miscarriages five in the first trimester, and two in the second trimester. Thus, the miscarriage rate was 5/31 (16.1%) among those who acquired the infection in the first trimester and 2/55 (3.6%) among those who acquired it in the second trimester. All women who had a miscarriage exhibited mild COVID‐19 disease. There were no pre‐existing risk factors associated with second‐trimester miscarriage. Moreover, a further nine women in the first and second trimester chose to undergo termination of pregnancy due to anxiety about potential adverse pregnancy outcomes caused by COVID‐19 infection in early pregnancy, or concerns about adverse effects from medication and radiological examinations.^33^, ^53^, ^62^

Overall, 485 (76.1%) of the 637 study women had delivered by the end of the study period, comprising 479 (98.6%) live births and 7 (1.4%) stillbirths, and 135 women were still pregnant. Six stillbirths occurred preterm between 24^+0^ and 30^+3^ gestational weeks.^15^, ^42^, ^59^ All these six stillbirths were among women with severe or critical COVID‐19 who were in the ICU on IMV; four women died after delivering a stillborn. One stillbirth occurred at term in a woman with a mild disease.^31^

Among the 479 women who had a live birth, there were 161 (33.7%) preterm deliveries (24^+0^–36^+6^ weeks) and 318 (66.4%) term deliveries (≥37 weeks).^5^, ^7^, ^10^, ^12^, ^15^, ^17^, ^18^, ^19^, ^20^, ^21^, ^22^, ^23^, ^25^, ^26^, ^28^, ^29^, ^31^, ^32^, ^33^, ^36^, ^37^, ^42^, ^44^, ^45^, ^46^, ^47^, ^48^, ^52^, ^55^, ^59^, ^60^, ^62^, ^66^ Overall, the mode of delivery was cesarean for 403 (84.1%) women and vaginal for 76 (15.9%) women. Of the 161 preterm deliveries, the exact gestational age was not specified in 42 cases^9^, ^18^, ^37^, ^52^ and the mode of delivery was not described for 35 preterm deliveries in two case series.^18^, ^37^ Thus, among the remaining 119 preterm deliveries where information pertaining to gestational age and mode of delivery were reported, there were 48 (40.3%) early preterm deliveries (24^+0^–33^+6^ weeks) and 71 (59.7%) late preterm deliveries (34^+0^–36^+6^ weeks).

Information on mode of delivery was provided in 126 preterm neonates; all were delivered by cesarean sections, accounting for 31.3% (126/403) of the total number of cesareans performed. Among 119 preterm deliveries where information about mode of delivery and disease severity were available, the indication for delivery was ‘maternal SARS‐CoV‐2 infection’ in 59/119 (49.6%) of preterm deliveries, involving 10 early and 49 late preterm pregnancies among women with mild symptoms and no fetal or maternal complications. The remaining 60/119 (50.4%) preterm deliveries were performed owing to medical indications to aid maternal resuscitation among those with severe or critical COVID‐19. Of these 60 women, 28 (46.7%) had evidence of concurrent fetal distress. Among the preterm deliveries, spontaneous preterm labor complicated 25 pregnancies (3.9% of all pregnancies exposed to SARS‐CoV‐2), of which 8 reported preterm prelabor rupture of membranes. The spectrum of severity of COVID‐19 among women who presented with spontaneous preterm labor included 14 (56.0%) mild and 11 (44.0%) severe or critical infections. All women who experienced preterm labor delivered by cesarean.

Among the 318 term neonates, 76 were delivered vaginally and 242 were delivered by cesarean. The indication for cesarean was deteriorating maternal respiratory condition for 25 women, all of whom had severe or critical disease; fetal distress for 22 women (9 with severe and 13 mild disease); failure to progress in labor for 19 women; previous cesarean for 15 women; prolonged rupture of membranes for one woman; and pre‐eclampsia for one woman. In 159 (65.7%) cases, cesarean was performed because of COVID‐19 among women with mild disease and no fetal and maternal compromise.

Adverse neonatal outcomes, including admission to the neonatal ICU (NICU), neonatal death rate, and vertical transmission were assessed among 479 live births; in addition, Apgar scores were reported in 29 studies comprising 361 newborns (Table 2).^4^, ^5^, ^9^, ^10^, ^16^, ^17^, ^18^, ^20^, ^24^, ^28^, ^29^, ^33^, ^36^, ^38^, ^39^, ^41^, ^44^, ^45^, ^46^, ^47^, ^48^, ^49^, ^50^, ^51^, ^55^, ^56^, ^62^, ^63^ Notably, there were six neonates with an Apgar scores of less than 7 at 1 and 5 minutes of life, all of whom were delivered preterm owing to fetal distress among mothers with critical COVID‐19.

**Table 2:** Adverse fetal and neonatal outcomes stratified by maternal disease severity.

Outcome	Total no. of cases	Maternal mild infection	Maternal severe/critical infection
Spontaneous preterm labor	25	14	11
Preterm delivery (<37 wk)	119	59	60
Fetal distress	50	22	28
Small for gestational age	6	6	0
Stillbirth	7	1	6
Low Apgar score (<7)	6	0	6
NICU admission	54	3	51
Neonatal infection^a^	8	3	5
Neonatal death	5	0	5

Abbreviation: NICU, neonatal intensive care unit.

^a^ Positive SARS‐CoV‐2 naso/ oropharyngeal swab.

Overall, 54 (11.3%) of newborns were admitted to the NICU. Of these, 3 were delivered by mothers with mild COVID‐19, and 51 were delivered by mothers with severe or critical infection.^45^, ^52^ Notably, 96.3% (52/54) of NICU admissions comprised preterm neonates. The two term neonates who were admitted to the NICU had neonatal respiratory distress and multicystic kidney disease, respectively.^12^ Other neonatal symptoms not requiring NICU admission but warranting admission to the special care unit included tachypnoea (n=11),^11^, ^12^, ^35^, ^37^ neonatal pneumonia (n=8),^12^, ^47^, ^51^, ^67^ fever (n=3),^37^, ^66^ rash (n=2),^35^ tachycardia (n=1), vomiting (n=1), and pneumothorax (n=1).^37^ All neonates admitted to the special care unit had negative nasopharyngeal COVID‐19 swabs.

Among the 479 live births, the incidence of neonatal death was 1.0% (n=5).^15^, ^21^, ^37^, ^44^ All five of these neonates were born to mothers with critical COVID‐19 admitted to the ICU. Notably, one neonate died on day nine of life owing to septic shock and multi‐organ failure consumptive coagulopathy after emergency delivery by cesarean at 34^+5^ gestational weeks. Another two deaths were twins who were delivered at 28 gestational weeks due to signs of fetal distress. They died on day three of life owing to complications of prematurity, despite normal blood parameters and chest radiographs. Their mothers also died from complications of critical COVID‐19 infection. The last two neonates were born with Apgar scores of 1 at 1 and 5 minutes after cesarean delivery due to signs of fetal distress among women with critical disease. The neonates died on days 0 and 1 of life, respectively. All five neonates who died had negative nasopharyngeal COVID‐19 swabs.

Of the 479 live newborns, 405 were tested for COVID‐19 via naso‐ and oropharyngeal swabs and 8 (2.0%) positive swabs were detected, obtained between 16 and 48 hours of life.^17^, ^18^, ^29^, ^50^ One of the positive swabs was a repeat test conducted 24 hours after a negative swab at the time of birth.^66^ Of those who tested positive, only one was asymptomatic^11^; the other seven neonates were admitted to the NICU with signs of chest infection. Among all neonates who tested positive for SARS‐CoV‐2, only one was delivered vaginally and was breastfed because maternal infection was diagnosed in the postpartum period.^50^ Body fluids that were analyzed to assess the risk of vertical transmission included amniotic fluid (n=20)^29^, ^50^, ^63^, ^66^ and umbilical cord blood (n=29) PCR for SARS‐CoV‐2,^24^, ^50^, ^63^, ^66^ which yielded negative results. Surface swabs were taken from six placentas,^24^, ^29^, ^43^, ^51^, ^54^ with only one positive result.^54^ Breast milk PCR testing was reported in five studies^8^, ^24^, ^43^, ^50^, ^51^, ^54^ (n=19), with only one positive test for the virus. Two studies reported reciprocal increased levels of maternal and neonatal IgG and IgM levels, as well as raised IL‐6 and white cell count in these newborns.^36^, ^38^

## 4 DISCUSSION

To our knowledge, the present review is one of the largest international systematic reviews of published studies on COVID‐19 infection in pregnancy, stratified by risk according to severity of COVID‐19 disease and timing in pregnancy. The review has assessed outcomes across a latitude of geographic locations, including Asia (mainly China), Europe, and the United States, from the start of epidemic in Hubei Province to its eventual reach as a global pandemic. The review provides sufficient data to support previous findings that pregnancy is not associated with a more severe course of infection overall. However, women in their third trimester and those with co‐morbidities are at particular risk of developing critical infection. This review also provides lessons from the high rate of obstetric interventions due to concerns of worsening maternal condition, potentially resulting both in cesaerean deliveries that are not indicated and in iatrogenic preterm birth with associated adverse neonatal outcomes among women with only mild COVID‐19.

Most pregnant women admitted to hospital had mild COVID‐19 with good outcomes, and only approximately 24% had severe or critical disease. The latter two subgroups accounted for the majority of adverse maternal, fetal, and neonatal outcomes. All maternal deaths, consumptive coagulopathies, and thrombotic complications occurred among women with severe and critical disease. Similarly, all instances of neonatal death, and NICU admissions and six of seven stillbirths occurred among neonates of mothers with severe or critical COVID‐19. These findings support a policy of safely managing pregnant women with mild infection in an outpatient setting to avoid unnecessary hospital admission during the flux of a pandemic with stretched healthcare services. Nevertheless, given that 3% of women with mild COVID‐19 in the present study deteriorated to a more severe infection, it is imperative that these women continue to be regularly reviewed and have access to acute medical care.

The review found that 9.6% of women were admitted to ICU and the overall maternal mortality was 1.6%. In a recent report from the US Center of Disease Control and Prevention of COVID‐19 among women aged 15–44years, 31.5% of pregnant women were hospitalized as compared with 5.8% of non‐pregnant women.^68^ After adjustment for cofounding factors, pregnant women were also more likely to be admitted to the ICU and receive mechanical ventilation, but there was a similar death rate of 0.2% among the two groups of women.^68^ However, data were not available to determine whether hospitalization and ICU admission were due to COVID‐19 or pregnancy‐related indications. A lower threshold for hospital admission in pregnancy may explain a higher rate of hospitalization; in turn, this may render a comparison of morbidity and mortality with a non‐pregnant population impossible because the majority of pregnant women admitted to hospital have mild disease, whereas individuals with COVID‐19 are generally admitted to hospital only with severe or critical infection. In the present review, the mortality rate among women with severe/critical COVID‐19 and those admitted to ICU was 6.7% and 16.4%, respectively. Women in their third trimester manifested the greatest need for ICU admission and IMV, and had the highest risk of death. Furthermore, there was a high incidence of key risk factors among pregnant women with poor maternal outcomes. The prevalence of obesity, diabetes mellitus, and advanced maternal age (≥40 years) was 40% among all maternal mortalities. Therefore, women with pre‐existing co‐morbidities are likely to be at highest risk of morbidity and mortality from SARS‐Cov‐2 infection in the third trimester, which warrants increased vigilance from healthcare providers.

COVID‐19 is a novel infection with rapidly evolving scientific data regarding its management. This has a significant impact on the psychosocial wellbeing of women during pregnancy. In a study from Ireland, 71 pregnant women expressed anxiety about the wellbeing of their family and 63% were concerned about the wellbeing of their unborn baby.^69^ In the present systematic review, there were nine cases of termination of pregnancy following SARS‐CoV‐2 infection in the first or second trimester owing to concerns about fetal development after maternal infection in early pregnancy. It is hoped that, as our understanding of COVID‐19 and its impact on pregnancy and the fetus evolves, we will be better informed to counsel women struggling with difficult decisions such as these.

Coagulopathy and thromboembolism are now recognized complications of severe COVID‐19 and are associated with poor outcomes.^70^, ^71^ A high rate of thrombotic events have been reported among non‐pregnant women hospitalized with COVID‐19, especially in those admitted to the ICU.^72^, ^73^, ^74^ It is known that the hypercoagulable state of pregnancy and puerperium predisposes women with sepsis to increased thromboembolic risk. In the review, only two cases of pulmonary embolism and one arterial line thrombosis were reported among pregnant women admitted to the ICU; this rate of 5% (3/61) is much lower than that reported in studies of non‐pregnant patients in the ICU with COVID‐19.^72^, ^73^, ^74^ In the latter studies, however, the majority of thrombotic events involved distal (segmental and sub‐segmental) pulmonary vessels, suggesting an underlying immune mechanism for thrombi within the lung rather than a true pulmonary embolism that is typically caused by pregnancy‐associated hypercoagulation and venous stasis. This may explain the lower rate of thromboembolism documented in the present review. Prospective data are required to assess the risk of coagulopathy, and the rate and type of thrombotic complications in COVID‐19 among pregnant and postnatal women.

There remains a paucity of data on the impact of COVID‐19 on pregnancy in the first and second trimester. The present review, in line with UK Obstetric Surveillance data,^75^ shows that women in early pregnancy account for a minority of hospitalized pregnant women with COVID‐19. Whether they are less likely to have severe infection, or whether clinicians have a lower threshold to admit women in the latter part of pregnancy is unknown. In the review, the rate of miscarriage was approximately 16% in first trimester and 4% in the second trimester among women with SARS‐CoV‐2 infection. The latter seems to be higher than the baseline abortion rate of 1%–2% in the second trimester.^76^, ^77^ However, the number of women with COVID‐19 during the second trimester of pregnancy reported in the review was small, and bias cannot be excluded because women with COVID‐19 who miscarry are potentially more likely to be reported in studies. It is important to ascertain the actual abortion rates among affected women with different disease severities. This will require the capture of data from women not admitted to hospital, which were not collected in the review. This is an area that requires further investigation to assess placental response, considering the multi‐organ impact of COVID‐19 and emerging evidence of its resemblance to complement‐mediated thrombotic microangiopathies.^78^ A recent study demonstrated at least one feature of maternal vascular malperfusion and intervillous thrombi among 15 placentae from women infected with COVID‐19.^79^ In the present review, placental histopathology in 14 cases revealed evidence of placental dysfunction in preterm placentae and in pregnancies with prepartum hemorrhage, second‐trimester miscarriage and small‐for‐gestational‐age newborns. This demonstrates the need for larger studies looking at the correlation between the degree of placental vascular thrombosis and adverse perinatal outcomes in COVID‐19.

The data reveal that a high proportion of severe (~60%) and critically unwell (~80%) pregnant women had both raised D‐dimer and raised IL‐6 levels, which are biomarkers of abnormal coagulation and molecular inflammation, respectively. This may have prognostic value in predicting COVID‐19 severity and poor outcomes, especially as these factors have been implicated in orchestrating the cytokine storm observed in critically unwell non‐pregnant patients with the infection.^80^ Data from non‐pregnant women demonstrate a strong association among elevated IL‐6 levels, risk of respiratory failure, and the subsequent need for mechanical ventilation.^81^ Although the present data highlight the prognostic utility of IL‐6 and D‐dimer assessment among pregnant women with COVID‐19, previous studies have reported fluctuations in these parameters in pregnancy.^82^, ^83^ This substantiates the need for future research to ascertain the normal ranges of these parameters in pregnancy in order to establish pregnancy‐adjusted thresholds for use in SARS‐CoV‐2 infection among women at different stages of pregnancy.

During the early surge of COVID‐19, there was a lack of information on the vertical transmission potential of SARS‐CoV‐2, resulting in understandable anxiety among women and obstetricians. This is reflected in the high rates of preterm birth (~34% of all births) and cesarean delivery in the review. Although deteriorating maternal condition and fetal distress accounted for ~50% of all preterm births, the remainder seemed to be iatrogenic among women with mild COVID‐19 with no maternal or fetal compromise. It seems unlikely that COVID‐19 itself increases the risk of spontaneous preterm labor; the rate in the review was approximately 4% (equally distributed among women with mild and severe/critical disease) as compared with a rate of 5%–8% reported for the general population.^84^ The overall high rate of preterm birth is likely to contribute to adverse neonatal outcomes and increased NICU admissions, leading to significant pressure on resource‐strapped neonatal units at the time of a pandemic.

The review demonstrated that COVID‐19 increases perinatal mortality with an overall stillbirth rate of 1.4% and neonatal death of 1.0%. Stillbirth and neonatal mortality rates are 0.42%^85^ and 0.28%^86^ in England and Wales, indicating a threefold increase in stillbirth and neonatal mortality among pregnancies affected by COVID‐19. All stillbirths (except one) and neonatal deaths were preterm and occurred among women with severe or critical disease, culminating in stillbirth and neonatal death rates of approximately 4% and 3%, respectively, among 150 women with severe or critical disease. This is comparable to respective rates of 3.6% and 5% reported in instances of severe maternal sepsis not due to COVID‐19.^87^

The possibility of vertical transmission of SARS‐CoV‐2 virus has been widely debated and the picture remains unclear. In the present study, only 8 (2%) of 405 neonates who underwent naso‐ and oropharyngeal PCR testing for SARS‐CoV‐2 virus had a positive test. Some neonates were also found to be positive for throat swabs immediately after cesarean delivery, despite measures to isolate the neonate from the mother on delivery. Recently, the visualization of SARS‐CoV‐2 virions in syncytiotrophoblasts and microvilli of the placenta suggests that the placenta, which is known to express the ACE2 receptor (the putative receptor for SARS‐CoV‐2), may be a target for virion entry.^88^ However, those findings are not confirmatory of vertical transmission.

A major strength of the review is the inclusion of data from a large number of pregnant women with laboratory‐confirmed SARS‐CoV‐2 infection from various regions of the world, including Asia, Europe, and the United States. However, the analysis also has limitations because COVID‐19 management decisions, as well as variations in healthcare resources, differ from country to country. In addition, datasets were obtained from retrospective observational studies that are prone to recall and/or misclassification bias, and limited the ability to explore risk factors. However, the overall risk for publication bias was moderate and more than 85% of the case series in the review adequately described the research questions and study population, and ensured that study women were comparable.

In conclusion, as our collective understanding grows, we believe that obstetricians will become less interventional, especially in preterm gestations, when managing mild COVID‐19 disease, thereby reducing the burden of iatrogenic neonatal morbidity. The data support previously documented findings of reassuring maternal outcomes for mild COVID‐19 infection, with poor maternal and fetal outcomes among those with severe or critical disease, predisposed by obesity, diabetes mellitus, advanced maternal age, and advanced pregnancy. This, we believe, should inform the counselling of women diagnosed with COVID‐19 in pregnancy.

## AUTHOR CONTRIBUTIONS

OT was responsible for conceptualization, project administration, investigation, methodology, validation, data curation, formal analysis, and drafting and revising the manuscript. AH was responsible for conceptualization, methodology, formal analysis, data curation, and drafting and revising the manuscript. PD was responsible for investigation, validation, and drafting and revising the manuscript. WJL was responsible for investigation, validation, and drafting and revising the manuscript. AW was responsible for and drafting and revising the manuscript. RA‐K was responsible for conceptualization, methodology, validation, supervision, and revision of the manuscript.

## CONFLICTS OF INTEREST

The authors have no conflicts of interest.

## Supporting information


**File S1**. Clinical characteristics of pregnant women at initial presentation of COVID‐19 disease.
