# Supplementary material for: Clinical characteristics, prognostic factors, and maternal and neonatal outcomes of SARS‐CoV‐2 infection among hospitalized pregnant women: A systematic review
Source: Int J Gynaecol Obstet. 2020 Aug 30;151(1):7–16. doi: 10.1002/ijgo.13329 (PMC9087651; doi:10.1002/ijgo.13329)
Supplement: Supplementary file 1 — File S1. Clinical characteristics of pregnant women at initial presentation of COVID‐19 disease. [file IJGO-151-7-s001.docx]

**Supplementary Materials**

**Supplementary File S1** Clinical characteristics of pregnant women at initial presentation of COVID-19 disease.

| Study | Study design | Sample size | Trimester at diagnosis (n) τ | | | COVID-19 Severity (n) | | | Presenting symptoms (n) | | | | | | | Blood results at presentation (n) |
| --- | --- | --- | --- | --- | --- | --- | --- | --- | --- | --- | --- | --- | --- | --- | --- | --- |
|  |  |  | **1** | **2** | **3** | **Mild** | **Severe** | **Critical** | **Fever** | **Cough** | **SOB** | **Myalgia/**  **Malaise** | **Diarrhoea** | **Asymptomatic** | **Other** |  |
| Dong L et al, 2020 | Case report | 1 |  |  | 1 | 1 |  |  | 1 |  |  |  |  |  |  | Raised IgG and IgM |
| Chen H et al, 2020 | Case series | 9 |  |  | 9 | 9 |  |  | 7 | 4 |  | 5 |  |  | Acute cholecystitis (1)  Sore throat (2) | Lymphopenia (5)  Transaminitis (3)  Raised CRP (7) |
| Chen Y et al, 2020 | Case series | 4 |  |  | 4 | 3 | 1* |  | 3 | 2 | 1 | 2 |  |  | Headache (1)  Reduced FM (1) | Lymphopenia (4)  Leukocytopenia (2)  Raised CRP (4) |
| Zhu H et al, 2020 | Case series | 9 |  |  | 9 | 9 |  |  | 9 | 9 |  |  | 1 | 1 | Acute cholecystitis (1) |  |
| Fan C et al, 2020 | Case series | 2 |  |  | 2 | 2 |  |  | 2 |  |  |  |  |  | APH (2)  Skin rash (1) |  |
| Chen S et al, 2020 | Case series | 3 |  |  | 3 | 3 |  |  | 3 |  |  |  |  |  |  |  |
| Liu D et al, 2020 | Case series | 15 | 1 | 3 | 11 | 15 |  |  | 13 | 9 | 1 | 7 | 1 |  | Sore throat (1) | Lymphopenia (12)  Raised CRP (10) |
| Zhang L et al, 2020 | RCS | 16 |  |  | 16 | 15 | 1 |  |  |  |  |  |  |  |  |  |
| Zeng H et al, 2020 | Case series | 6 |  |  | 6 | 6 |  |  | ‘Mild symptoms’ | | | | | | | |
| Yu N et al, 2020 | Case series | 7 |  |  | 7 | 7 |  |  | 6 |  | 1 |  | 1 |  |  | Lymphopenia (5)  Neutrophilia (5)  Raised D-Dimer (7)  Transaminitis (2)  Raised IL-6 (4)  Raised procalcitonin (1)  Raised CRP (7) |
| Li Y et al, 2020 | Case report | 1 |  |  | 1 | 1 |  |  | 1 | 1 |  |  |  |  |  |  |
| Liu Y et al, 2020 | Case series | 13 |  | 2 | 11 | 12 |  | 1 | 10 | 3 |  | 10 |  |  |  |  |
| Lee DH et al, 2020 | Case report | 1 |  |  | 1 | 1 |  |  |  |  |  |  |  |  |  |  |
| Xiong X et al, 2020 | Case report | 1 |  |  | 1 | 1 |  |  | 1 | 1 |  |  |  |  |  | Lymphopenia |
| Wu X et al, 2020 | RCS | 23 |  |  | 23 | 23 |  |  |  |  |  |  |  | 15 |  |  |
| Wen R et al, 2020 | Case report | 1 |  |  | 1 | 1 |  |  |  |  |  |  | 1 |  |  |  |
| Breslin et al, 2020 | RCS | 43 |  |  | 43 | 37 | 4 | 2 | 14 | 19 | 7 | 11 |  | 14 |  |  |
| Karami P et al, 2020 | Case report | 1 |  |  | 1 |  |  | 1 | 1 | 1 | 1 | 1 |  |  |  | Leukopenia  Thrombocytopenia  Raised LDH  Prolonged aPTT & PT  Raised D-Dimer  Raised CRP |
| Khan S et al, 2020 | Case series | 17 |  |  | 17 | 17 |  |  | 3 | 6 | 2 |  | 3 |  | Congestion (2) | Leukocytosis (8)  Normal or low leukocytes (9)  Lymphopenia (4)  Transaminitis (2) |
| Zhou R et al, 2020 | Case report | 1 |  |  | 1 | 1 |  |  |  |  |  |  |  | 1 |  | Normal WCC, U&E, LFT, coagulation screen, D-Dimer and Procalcitonin levels |
| Chen L et al, 2020 | Case series | 84 | 16 | 18 | 50 | 74 | 9 | 1 | 61 | 57 | 8 | 6 |  | 6 | N&V (4)  Chest pain (4)  Sore throat (2)  Headache (7) | Leukopenia (3)  Leukocytosis (16)  Lymphopenia (40)  Thrombocytopenia (2)  Normal fibrinogen  Transaminitis (15)  Raised D-Dimers (59)  Raised procalcitonin (9)  Raised CRP (47) |
| Li Y et al, 2020 | Case report | 1 |  |  | 1 | 1 |  |  |  |  |  |  |  | 1 |  | Normal WCC, LFT, U&E and CRP |
| Liu Y et al, 2020 | Case report | 1 |  |  | 1 | 1 |  |  |  |  |  |  | 1 |  | Nausea (1) | Normal WCC, LFT, U&E and Coagulation screen  Raised CRP |
| Zhao R et al, 2020 | Case report | 1 |  | 1 |  |  | 1 |  | 1 | 1 |  |  |  |  |  | Raised LDH  Normal WCC  Raised CRP |
| Chen X et al, 2020 | Case series | 3 | 1 | 1 | 1 | 2 | 1 |  | 3 |  | 1 |  | 1 |  |  | Raised ESR (3)  Raised CRP (3)  Raised IL-6 &10 (3)  Normal lymphocytes, U&E and LFT |
| Yu N et al, 2020 | Case report | 1 | 1 |  |  | 1 |  |  | 1 |  |  | 1 | 1 |  |  |  |
| Wu C et al, 2020 | Case series | 6 |  |  | 6 | 6 |  |  |  |  |  |  |  | 6 |  | Post-partum lymphopenia (5)  Raised post-partum WCC (5), particularly higher in 2 women who developed pyrexia  Raised D-Dimer (5)  Raised CK (4)  Normal LFT  Raised CRP (6) |
| Browne PC et al, 2020 | Case report | 1 |  | 1 |  | 1 |  |  | 1 | 1 |  |  |  |  |  | Raised WCC |
| Xia H et al, 2020 | Case report | 1 |  |  | 1 |  | 1* |  | 1 | 1 |  |  |  |  |  | Lymphopenia  Low WCC  LFT, U&E |
| Hantoushzadeh S et al 2020 | Case series | 9 |  | 2 | 7 |  | 9 |  | 9 | 8 | 7 | 4 |  |  | Sore throat (1)  PPROM (1)  Reduced FM (1) | Lymphopenia (2)  Pancytopenia (1)  (Blood results for 7 women not reported) |
| Baud D et al, 2020 | Case report | 1 |  | 1 |  | 1 |  |  | 1 | 1 |  | 1 | 1 |  | Sore throat (1)  Contractions (1) |  |
| Hu X et al, 2020 | Case series | 7 |  |  | 7 | 7 |  |  | 4 | 2 |  |  | 1 |  |  |  |
| Zamaniyan M et al, 2020 | Case report | 1 |  |  | 1 |  | 1 |  | 1 | 1 | 1 | 1 |  |  | Nausea (1)  Anorexia (1) | Lymphopenia (1)  Raised CRP (1) |
| Alzamora M et al, 2020 | Case report | 1 |  |  | 1 |  | 1 |  | 1 | 1 |  | 1 |  |  |  | Pancytopenia (1)  Raised CRP (1)  Raised Ferritin (1)  Raised D-Dimer (1) |
| Pierce-Wiliams RAM et al, 2020 | Case series | 64 |  | 10 | 54 |  | 44 | 20 |  |  |  |  |  |  |  | Raised IL-6, ferritin (64)  Transaminitis (64)  Raised D-Dimers, procalcitonin (64)  Raised troponin,LDH, CPK (20)  Raised CRP (64) |
| Zambrano LI et al, 2020 | Case report | 1 |  |  | 1 | 1 |  |  | 1 | 1 |  | 1 |  |  |  |  |
| Vlachodimitropoulou KE et al, 2020 | Case series | 2 |  |  | 2 | 2 |  |  | 2 | 2 |  |  |  |  |  | Thrombocytopenia (2)  Abnormal clotting screen (2)  Reduced fibrinogen (2) |
| Yan J et al, 2020 | Case series | 65 | 4 | 6 | 55 | 59 | 6 |  | 45 | 28 | 11 | 18 | 1 | 6 | Sore throat (10) | Leucopenia (20=  Leucocytosis (3)  Lymphoenia (38)  Lymphocytosis 1)  Raised CRP (32) |
| Ferazzi E et al, 2020 | Case series | 42 |  |  | 42 | 31 | 7 | 4 | 26 | 18 | 8 |  | 2 |  |  | Leucocytosis (16)  Lymphopenia (6)  Raised CRP (17)  Transaminitis (5) |
| Gidlöf S et al, 2020 | Case report | 1 |  |  | 1 |  | 1* |  |  |  |  | 1 | 1 |  | Headache (1)  Hoarseness (1) |  |
| Iqbal S et al, 2020 | Case report | 1 |  |  | 1 | 1 |  |  | 1 | 1 |  | 1 |  |  | Reduced FM (1) | Lymphopenia (1) |
| Juusela A et al, 2020 | Case series | 2 |  |  | 2 |  | 2* |  | 2 |  |  |  |  |  |  |  |
| Kalafat E et al, 2020 | Case report | 1 |  |  | 1 |  |  | 1** |  | 1 | 1 |  |  |  | Reduced FM (1) |  |
| Kelly J C et al, 2020 | Case report | 1 |  |  | 1 |  |  | 1** | 1 | 1 |  |  |  |  |  | Lymphopenia (1)  Tansaminitis (1) |
| Schnettler W T et al, 2020 | Case report | 1 |  |  | 1 |  |  | 1** | 1 | 1 | 1 | 1 |  |  |  | Leucopenia (1)  Lymphopenia (1)  Thrombocytopenia (1)  Transaminitis (1)  Raised prolactin (1) |
| Lu D et al, 2020 | Case report | 1 |  |  | 1 | 1 |  |  |  |  |  |  |  | 1 |  | All blood results normal |
| Khan S et al, 2020 | Case series | 3 |  |  | 3 | 3 |  |  | 1 | 1 | 1 |  |  |  | Chest tightness (1) | Raised CRP (2) |
| Hong L et al, 2020 | Case report | 1 |  | 1 |  |  | 1* |  | 1 | 1 | 1 | 1 |  |  |  | Raised CRP  Lymphocytopenia  Transaminitis  Raised CPK |
| Li J et al, 2020 | Case report | 1 |  |  | 1 |  |  | 1** | 1 | 1 | 1 |  |  |  | Sore throat | Lymphopenia  Thrombocytopenia  Abnormal clotting screen  Raised IL-6, procalcitonin and CRP |
| Kuhrt K et al, 2020 | Case report | 1 |  |  | 1 | 1 |  |  | 1 | 1 | 1 | 1 |  |  | APH | Lymphopenia  Thrombocytopenia |
| Wu Y et al, 2020 | Case series | 13 | 5 | 3 | 5 | 13 |  |  |  |  |  |  |  |  |  |  |
| Baergen R N et al, 2020 | Case series | 20 |  |  | 20 | 20 |  |  | 2 |  | 2 |  |  | 16 |  |  |
| Rosen M H et al, 2020 | Case report | 1 | 1 |  |  | 1 |  |  |  |  |  |  |  |  | Flare up of UC | Raised CRP  Normal D-dimer  Normal troponin |
| Ahmed I et al, 2020 | Case report | 1 |  |  | 1 |  |  | 1** | 1 |  | 1 |  |  |  | Diabetic ketoacidosis |  |
| London V et al, 2020 | Case series | 68 |  | 3 | 65 | 68 |  |  | 18 | 29 |  |  |  |  |  |  |
| Lokken EM et al, 2020 | Case series | 8 |  |  | 8 |  | 8 |  |  |  |  |  |  |  |  |  |
| Anderson J et al, 2020 | Case report | 1 |  | 1 |  |  |  | 1** | 1 | 1 | 1 | 1 |  |  |  |  |
| Cooke WR et al, 2020 | Case series | 2 |  |  | 2 |  | 1 | 1** | 2 |  | 2 | 2 | 2 |  |  |  |
| Xiaotong W et al, 2020 | Case report | 1 |  |  | 1 | 1 |  |  | 1 |  |  |  |  |  |  | Raised CRP  Raised procalcitonin  Raised D-dimer  Raised CK |
| Xu Q et al, 2020 | RCS | 28 | 3 | 1 | 24 | 26 | 2 |  | 5 | 7 | 2 | 1 |  |  | Abdominal pain (5) | Leucocytosis (10)  Raised CRP (17)  Raised LDH (4)  Raised CK |
| Govind A et al, 2020 | Case series | 2 |  | 1 | 1 |  |  | 2** | 1 | 2 |  | 1 |  |  | Chest pain (1) | Lymphopenia (2) |
| Collin J et al, 2020 | Case series | 11 |  |  |  |  |  | 11 |  |  |  |  |  |  |  |  |
| Martinelli et al, 2020 | Case report | 1 |  |  | 1 | 1 |  |  | 1 |  | 1 |  |  |  | Rhinitis | Raised CRP  Raised D-dimer |
| Total |  | 637 | 32 | 55 | 539 | 487 | 101 | 49 | 273 | 224 | 64 | 79 | 18 | 68 |  |  |

APH: Antepartum hemorrhage; APTT: activated partial thromboplastin time; CK: Creatine kinase; FM: Fetal movement; LFT: Liver function test; N&V: Nausea and vomiting; PPROM: Preterm premature rupture of membranes; PT: Prothrombin time; U&E: Urea and electrolytes; WCC: White cell count. *= Mild disease at presentation progression to severe disease **= mild at presentation, progression to critical disease

τ: Trimesters reported in 61 studies
